# Intratympanic injection of hydrogel nanodrug for the prevention and treatment of sensorineural hearing loss

**DOI:** 10.1016/j.joto.2023.09.005

**Published:** 2023-09-19

**Authors:** Tianying Zhai, Pingping Ai, Zhaohui Tang, Chaoliang He, Xuesi Chen, Shiming Yang, Nan Wu

**Affiliations:** a Senior Department of Otolaryngology-Head & Neck Surgery, PLA General Hospital, Beijing, China; b National Clinical Research Center for Otolaryngologic Disease, Beijing, China; c State Key Lab of Hearing Science, Ministry of Education, Beijing, China; d Beijing Key Lab of Hearing Impairment Prevention & Treatment, Beijing, China; e Zhejiang Chinese Medical University, Hangzhou, China; f Changchun Institute of Applied Chemistry, Chinese Academy of Sciences, Changchun, China

**Keywords:** Thermo-sensitive hydrogel, Nanodrug, Sensorineural hearing loss, Intratympanic injection

## Abstract

Safe and efficient drug delivery to the inner ear has always been the focus of prevention and treatment of sensorineural deafness. The rapid development of nanodrug delivery systems based on hydrogel has provided a new opportunity. Among them, thermo-sensitive hydrogels promote the development of new dosage form for intratympanic injection. This smart biomaterial could transform to semisolid phase when the temperature increased. Thermo-sensitive hydrogel nanodrug delivery system is expected to achieve safe, efficient, and sustained inner ear drug administration. This article introduces the key techniques and the latest progress in this field.

Safe and efficient drug delivery to the inner ear has always been the focus of acute sensorineural deafness treatment. Although guidelines recommend the oral and intravenous infusion of glucocorticoids, systemic administration inevitably results in insufficient drug concentration in the inner ear and excessive side reactions. Intratympanic injection allows drugs to directly enter the inner ear through the round window membrane (RWM), achieving high drug delivery efficiency and minimize systemic side effects. Hence, this technique has great clinical application prospects. However, drugs are discharged from the Eustachian tube after intratympanic injection, and the residence time in the middle ear is remarkably shortened. This phenomenon reduces patient compliance and increases the incidence of adverse reactions, such as tympanic membrane perforation.

The rapid development of nanodrug delivery systems has provided new opportunities for inner ear drug delivery. Among these systems, hydrogels can reversibly block the eustachian tube, prolong the residence time of drugs in the middle ear, and significantly improve the drug delivery efficiency to the inner ear by intratympanic injection. Thermo-sensitive hydrogels promote the development of intratympanic drug injection. This smart biomaterial is in the aqueous phase at a low temperature. As the temperature increases, a phase transition occurs. After the transition, a semisolid hydrogel is formed. This feature is conducive to the development of new drug dosage form for intratympanic injection. Hydrogel nanodrug delivery system can achieve safe, efficient, and sustained inner ear drug delivery through intratympanic injection.

On this basis, several research groups combined highly modifiable drug-loaded nanoparticles with hydrogel technology to construct a composite drug delivery system for tympanic injection. For example, a recent report focused on targeted drug-loaded nanoparticles to achieve targeted drug delivery to inner ear hair cells. Some teams have used physical or chemical methods to increase the permeability of RWM to improve the delivery efficiency.

In conclusion, the development of hydrogel-based nanodrug delivery system for intratympanic injection has made substantial progress. Some international research teams have promoted it into the clinical research. This article introduces the key techniques and the latest progress in this field, which was summarized in the following diagram (Fig. 1).

**Fig. 1:**
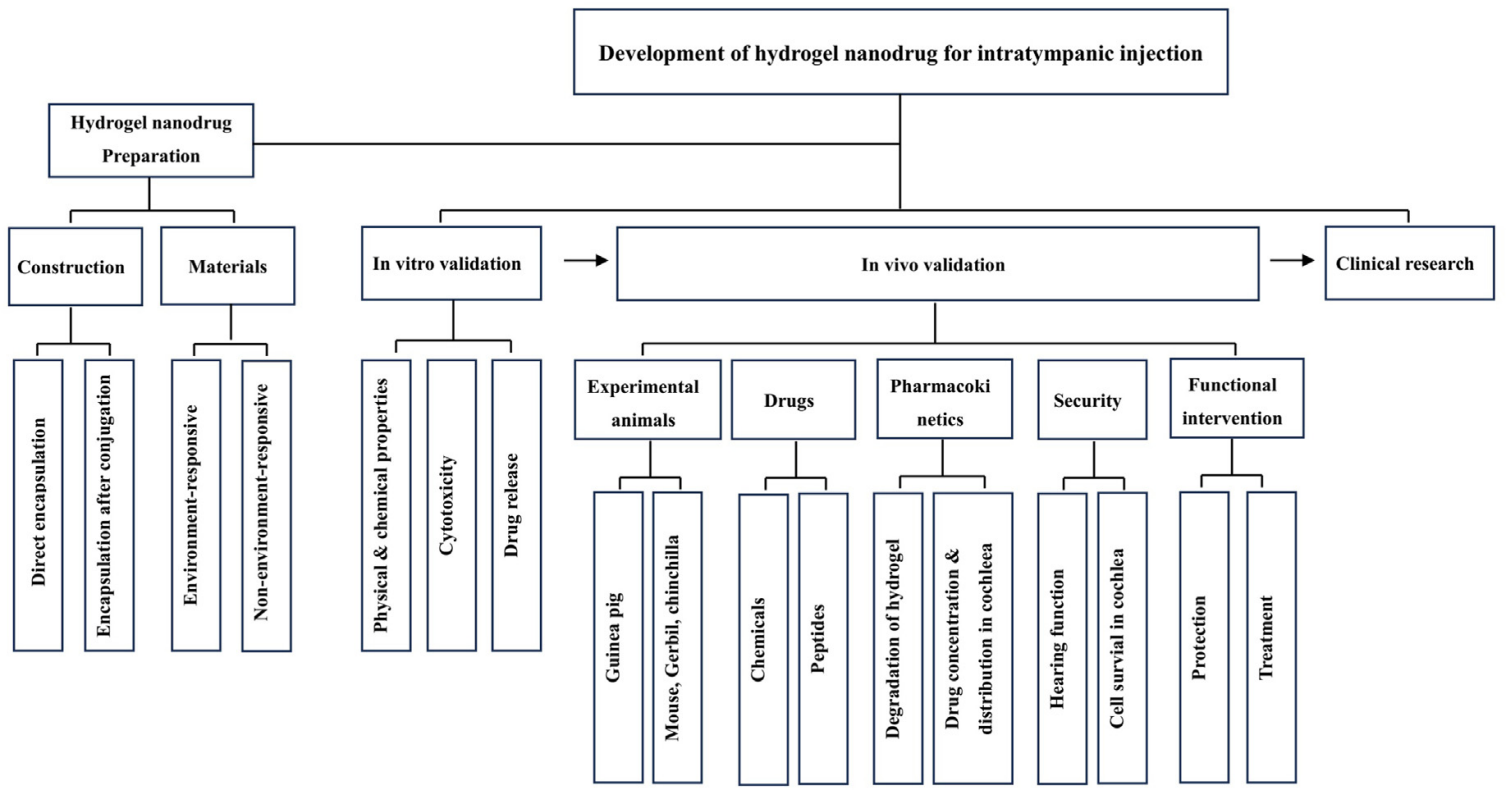
The diagram of article.

## 1 Construction and materials of hydrogel drug delivery system

The hydrogel drug delivery system for intratympanic injection can be constructed either by the direct encapsulation of the drug within the hydrogel or the conjugation of the drug with nanocarriers prior to encapsulation within the hydrogel. In light of hydrogels’ physical and chemical properties, they can be classified as either non-environment-responsive or environment-responsive, with the latter category including thermo-sensitive and photo-sensitive hydrogels.

The following are the hydrogel biomaterials most commonly employed in existing studies for drug delivery systems that involve the direct encapsulation of the drug within the hydrogel: gelatin hydrogel (Inaoka et al., 2009), hyaluronic acid hydrogel (Xu et al., 2018), silk-polyethylene glycol (Yu et al., 2016), diltiazem-chitosan hydrogel (Naples et al., 2020), and poloxamer 407 (Ahmadi et al., 2019) (POX407O). The first three serves as nonsmart responsive materials, and the latter two can be engineered into environment-responsive hydrogels with thermo-sensitive properties through specific cross-linking reactions or polymerization reactions.

A trending area of research in drug delivery systems is the development of composite drug delivery systems by combining drugs with nanocarriers and hydrogels. The main biomaterials employed in the corresponding studies were as follows: drug delivery systems studied after physical mixing, such as chitosan/poloxamer 407, and nanodrug-loaded particles (Honeder et al., 2014). Another investigation approach in drug delivery system involves cross-linking the hydrogel first and then incorporating nanoparticles to form an embedded drug delivery system, such as chitosan and glycerophosphate/thiol-ethylene cross-linking, hyaluronic acid, and 1,4-butanediol diglycidyl ether cross-linking, followed by embedding or other forms of carrying nanodrug-loaded particles for drug delivery (Xu et al., 2018). Laboratory investigations demonstrated that hydrogel-based composite drug delivery systems can achieve promising results in drug delivery and sustained release capabilities. Compared with the construction method of directly encapsulating drugs in hydrogel, such composite drug delivery system has exhibited superior drug dispersion and delivery repeatability and avoided degradation by in vivo biological enzymes. By modifying drug-loaded nanoparticles with targeted short peptides (Kayyali et al., 2018) that specifically bind to the prestin protein, a research team attained successful drug delivery to the inner ear hair cells using the chitosan/glycerophosphate hydrogel drug delivery system. Targeted delivery further enhances the clinical application prospect of the hydrogel drug delivery system for the inner ear.

The success of the aforementioned drug delivery system heavily relies on the material properties of the hydrogel, which can be altered by adding additives, changing the type of cross-linking compound, or adjusting the hydrogel content to ultimately affect the drug delivery efficiency to the inner ear. For example, in a study utilizing a PLGA–PEG–PLGA thermo-sensitive hydrogel drug delivery system, the addition of glycerol and poloxamer facilitated the phase transition time and drug release rate of the hydrogel in the middle ear cavity (Feng et al., 2014). An elevation in the former may expedite the deterioration of the hydrogel, curtail the drug's dwelling period in the middle ear cavity, and ultimately reduce the drug concentration that enters the inner ear. Conversely, the latter can bring about an opposite outcome. Unfortunately, a large number of similar studies solely focused on the properties and improvements of the materials but lacked evaluation of its in vivo application effects (Wang et al., 2021).

## 2 In vitro validation for the functional identification of hydrogel drug delivery systems

The in vitro verification of hydrogel drug delivery systems involves the identification of the physical and chemical properties of biological materials and the screening and identification of the biosafety and drug delivery efficiency of the drug delivery system using in vitro cultured cells.

The identification of physical and chemical properties of materials involves various techniques, including scanning electron microscope observation to determine the physical properties of the material such as particle size and dispersion coefficient (Yuksel Aslier et al., 2019) or to determine whether the embedding or cross-linking of the drug is successful, and carbon nuclear magnetic resonance and hydrogen spectra are used to verify the binding mode and crosslinking coefficient of the compound crosslinked material (Ju et al., 2020). Fourier transform infrared spectroscopy and differential scanning calorimetry often serve as auxiliary verification methods to evaluate compound interaction (Yuksel Aslier et al., 2019). Following drug binding or embedding, the hydrogel drug delivery system can be evaluated using high-performance liquid chromatography coupled with mass spectrometry to determine the success of cross-linking or embedding based on the expected type and quantity of absorption peaks observed (Yu et al., 2016). Emulsion cross-linking assays are employed to determine the drug encapsulation efficiency of hydrogel delivery systems (Li et al., 2020a). In the investigation of the in vitro drug release properties of hydrogel drug delivery systems, Franz diffusion cells are the go-to method and can also be utilized for verifying osmosis (Kim et al., 2021). Experiments such as gel transition experiments (temperature ramp experiments) and fluid characterization experiments are commonly used to identify the phase transition capabilities of materials with environmental response characteristics, such as thermo-sensitive hydrogels and photo-sensitive hydrogels. For instance, a temperature ramp experiment was performed to observe the phase transition time of a material from liquid to solid at different temperatures (Zhang et al., 2021).

The OC1 cell line is the most commonly selected for in vitro drug delivery studies on the auditory system (Yuksel Aslier et al., 2019; Chen et al., 2019). The L929 fibroblast cell line and SK-MEL-31 cell line have also been adopted in some works (Xu et al., 2018). In vitro research primarily involves measuring the cytotoxicity of biological materials by observing the activity of co-incubated cells; this cytotoxicity reflects the biocompatibility and application safety of the drug delivery system. MTT staining and CCK8 staining are the most commonly used research methods (Chen et al., 2022). The majority of hydrogel drug delivery systems have demonstrated low cytotoxicity when evaluated using the aforementioned research methods (Chen et al., 2022). Nevertheless, the cytotoxicity of biological materials can be accurately measured by flow cytometry for the determination of cellular reactive oxygen species. Furthermore, flow cytometry provides an accurate quantification of the cellular uptake rate of drug-loaded nanoparticles. For example, OC1 cells were employed to analyze the uptake of fluorescently conjugated multifunctional nanomaterials. Cell fluorescence was analyzed using flow cytometry (Luo and Xu, 2012). In Cy5-conjugated targeted multifunctional nanoparticle (tMFNP) nanodrug delivery system, tMFNP served as the additive with the hair cell-specific targeting modification protein PrTP1. The uptake of tMFNP after specific targeting modification was markedly higher than that of nontargeting MFNPs, verifying that the modification of PrTP1 enhanced the binding efficiency of MFNPs to these cells.

## 3 In vivo validation of hydrogel drug delivery systems

### 3.1 Types of experimental animals used in the study

Although guinea pigs are often used in research, their thin cochlear bones can lead to nonspecific drug penetration, making it difficult to accurately evaluate the pharmacokinetics of inner ear drug delivery through tympanic injection (Luo and Xu, 2012). Some investigations employed CBA mice, C57BL/6 mice, Mongolian gerbils, and chinchillas as animal models for research purposes. For the study of the in vivo delivery and distribution of fluorescently labeled drugs by in vivo imaging technology, nude mice fulfilled the demands of experimental observation (Ju et al., 2020).

### 3.2 Types of drugs used in the study

Hydrogel-based drug delivery systems have also been explored for the treatment of otitis media, a common inflammatory condition of the middle ear. Nevertheless, the majority of research still highlighted the development of biomaterials for drug delivery systems. The application of these systems in treating auditory diseases and enhancing auditory function is still in its early stages, with limited research available. Drugs for inner ear delivery are mainly categorized into two groups: chemical drugs and peptide-based biological drugs. Significant research and development have focused on chemical drugs, primarily on glucocorticoids (dexamethasone (Borden et al., 2011), triamcinolone acetonide (Zhu et al., 2018)), and gentamicin (Xu et al., 2010), and chemical drugs (including calcium channel blockers (Naples et al., 2020)). Relatively few studies employed peptide biopharmaceuticals, including recombinant human insulin-like growth factors (Fujiwara et al., 2008) (insulin-like growth factor-1, IGF-1) and various nerve growth factors (such as neurotrophin-3 (*9*) and heparin growth factor (Inaoka et al., 2009)). Peptide biological drugs that have been investigated for inner ear delivery include neurotrophic factors, cell-penetrating peptides, and antisense oligonucleotides. Histological analysis unfolded the survival of inner hair cells in animals treated with IGF-1 via hydrogel after 7 days of inner ear ischemia rate, putting forward the potential of hydrogel-delivered IGF-1 to protect the cochlea from ischemic injury (Fujiwara et al., 2008). In a different study, the use of sticky microspheres prolonged the residence time of Ebselen in the middle ear cavity for more than 7 days (Chen et al., 2022), resulting in a significant recovery of hearing in noise-exposed mice. On the 14th day after noise exposure, the hearing of each frequency returned to baseline levels. At 32 kHz, the outer hair cell survival recovered from 48% ± 6%–93% ± 2% (Chen et al., 2022) (Chen et al., 2022). Building on the PEG hydrogel cross-linked by thiol-vinyl sulfone Michael addition, it was modified with neurotrophin-3 (NT-3) nerve growth factor-specific affinity peptide and carried the therapeutic polypeptide. The duration of NT-3 nerve growth factor delivery to the inner ear was significantly extended by this drug delivery system compared with a poloxamer hydrogel (Wang et al., 2021).

### 3.3 Pharmacokinetic assessment

Hydrogels remain in the middle ear cavity (around the round window niche) for 7–14 days after intratympanic injection until they degrade completely (Honeder et al., 2014; Chen et al., 2022). The degradation rate of hydrogel can be determined by measuring and weighing the remaining hydrogel in the middle ear cavity after a certain period. Some research groups applied fluorescent dyes to label nanohydrogel drug delivery systems and inject them into the middle ear cavity. The speculum visualizes, samples, and counts intratympanic fluorescence intensity through the intact tympanic membrane to indirectly assess drug residence time in the middle ear cavity (Ju et al., 2020). Another method for monitoring the distribution and clearance of hydrogel drug delivery systems in the middle ear is through fluorescence imaging, which uses fluorescent dyes to label the hydrogel and track its movement in real time. However, the hydrogel drug delivery system is cleared from the middle ear cavity within 14 days after injection. (Videhult Pierre et al., 2019). Some hydrogel biomaterials, such as chitosan/phosphoglycerate hydrogel, boast a long residence time in the middle ear cavity due to their slow degradation rate (Kayyali et al., 2018; Lajud et al., 2015). To prevent the development of secretory otitis media resulting from prolonged Eustachian tube obstruction, researchers devised an additional injection of chitosanase to expedite in vivo degradation (Li et al., 2017).

High-performance liquid chromatography and mass spectrometry are the commonly used analytical techniques in the pharmacokinetic studies of intratympanic drug administration in the inner ear, with the inner ear lymph fluid being the main sample collected for analysis. Simultaneous measurement of drug concentrations in blood and CSF can be applied to assess systemic drug distribution throughout the body. Several studies disclosed that following the intratympanic injection of a hydrogel delivery system, drugs can penetrate the inner ear within 30 min to 24 h and remain at above therapeutic concentrations for 3–10 days (Fernandez et al., 2016). A research utilizing PLGA nanoparticles delivery system revealed that drugs could enter the inner ear within 24 h and remain above therapeutic concentrations for up to 7 days (Li et al., 2020b). When investigating the pharmacokinetics of dexamethasone in the inner ear, researchers observed an initial concentration of 1.18 ± 0.335 μg/ml in the inner ear lymph fluid and found that effective therapeutic concentrations were maintained for a minimum of 7 days. After injection, the initial drug concentration in the inner ear lymph is only 0.58 ± 0.21 μg/ml, which can only be maintained for 1–3 days. A different study demonstrated that an inner ear delivery system based on silk protein polyethylene glycol (Silk PEG) hydrogel could maintain the concentration of dexamethasone phosphate in the inner ear lymph fluid at a minimum of 100 ng/ml for 10 days. Nevertheless, dexamethasone phosphate was infused into the tympanum. By contrast, the control group demonstrated that clinically effective drug concentrations could only be sustained for less than 12 h (Yu et al., 2016). Although the gel drug delivery system can deliver drugs efficiently and consistently from the middle ear cavity to the inner ear, other studies proposed that after the drug enters the inner ear, it is mainly concentrated in the basal gyrus of the cochlea and cannot enter the middle parietal gyrus. Even when it entered, the gel drug is easily cleared in a short time (Naples et al., 2020).

The hydrogel drug delivery system can also fulfill the demands of precise drug delivery to the inner ear. For instance, intratympanic gentamicin serves as an alternative option for the treatment of refractory Meniere's disease. However, conventional drug delivery methods may lead to nonselective gentamicin ototoxicity in hair cells of cochlear and vestibular systems, and the severity of such damage may vary depending on the degree of drug end-organ absorption. (Li et al., 2020a). Therefore, achieving selective vestibular ablation and reducing cochlear hair cell damage are the key research focus for gentamicin injection into the tympanic cavity, with various administration protocols being investigated. Chitosan–glycerol–phosphate hydrogel embedded with 40 μg of gentamicin can control vertigo after intratympanic administration while maximally retaining auditory function, indicating its superiority to traditional gentamicin in the tympanic cavity. One advantage of the injection method is its superior effectiveness and safety in stark contrast to other methods of drug delivery (Xu et al., 2010).

### 3.4 Security

The potential of hydrogel drug delivery systems for inner ear drug delivery is contingent on their safety profile, as is the case with all drug delivery systems. Hydrogel's influence on the ossicular chain's activity results in low-frequency hearing loss, but this effect is only temporary. Thresholds of auditory brainstem responses were slightly elevated by day 3 post-dose and almost fully recovered by day 10 (*6*). Some studies analyzed the morphology of the mucosal epithelial cells in the middle ear to investigate whether drug administration via the tympanic membrane causes inflammation. Others evaluated inner ear cytotoxicity through morphological observation and inner ear hair cell counts. The majority of studies indicated that hydrogel delivery systems administered through the tympanic membrane route with various materials are safe for use (Naples et al., 2020; Salt et al., 2011).

### 3.5 Functional intervention

The objective of using hydrogel drug delivery systems in tympanic membrane injection for inner ear drug delivery is to protect or treat damaged inner ear function. Although the preparation and delivery of drug delivery systems have been widely studied, research on their actual therapeutic benefits for inner ear function is relatively limited. Only a few reports are available on the intervention of inner ear function. To a certain extent, this situation tell us that the research on the application of hydrogel nanodrug delivery system in ear diseases is still in its early development. According to published reports, auditory function is majorly measured by auditory brainstem response threshold and distortion of otoacoustic emissions, and inner ear hair cell and spiral ganglion cell counts are commonly used as morphological indicators of inner ear damage and recovery. Based on the type of intervention, research on hearing impairment can be classified as preventive or therapeutic. Meanwhile, based on the mechanism of hearing impairment of the intervention target, it can be categorized as research on noise-induced hearing loss, drug-induced hearing loss, or sudden hearing loss.

The chitosan glycerophosphate hydrogel nanodrug delivery system successfully carried diltiazem for inner ear delivery; cisplatin-induced ototoxicity in inner ear hair cell synapses was effectively reduced through the protective effect of diltiazem (Naples et al., 2020). In another study, a silk protein hydrogel drug delivery system carrying dexamethasone for inner ear delivery achieved remarkable hearing protection in cisplatin-induced deaf mice (Chen et al., 2019). For the treatment of acute inner ear ischemia, the intratympanic administration of gel containing IGF-1 30 min following acute inner ear ischemia can effectively promote the survival rate of inner ear hair cells and evidently improve hearing (Fujiwara et al., 2008).

## 4 Clinical research progress

In view of the huge clinical application potential of the hydrogel drug delivery system, some organizations have applied it for clinical research. In 2014, Kyoto University in Japan put forward a series of clinical studies based on the intratympanic injection of gelatin hydrogel. A hydrogel was adopted to treat refractory sudden deafness by injecting IGF-1 into the tympanic membrane. However, the findings revealed that the mean pure-tone hearing threshold between treatment groups varied over time in repeated-measures linearity. A mixed-model analysis failed to demonstrate the superiority of topical IGF-1 over intratympanic dexamethasone for the treatment of deafness, despite statistically significant differences over time. Otonomy, Inc. Has concluded a phase 3 clinical study of dexamethasone-loaded poloxamer gel extended-release formulation (OTVIDEXTM) (Nakagawa et al., 2014). The AVERTS-2 trial conducted in Europe in 2017 revealed the promising clinical efficacy of OTIVEXTM in patients with Meniere's disease, in accordance with an announcement made by the company. However, the previous AVERTS-1 trial conducted in the United States did not pose any striking results. Moreover, two hyaluronic acid hydrogels (AM-101 and AM-111) developed by Aurui Medical have ventured to phase 3 clinical development. Developed specifically for the treatment of acute tinnitus, AM-101 contains Estamine hydrochloride. Meanwhile, AM-111 contains bromapetin (D-JNKI-1) and is used to treat sudden hearing loss. Numerous European research and development institutions jointly published some findings of the phase 3 clinical study based on the above drug, concluding that though the primary endpoint was ineffective in the overall study population, a positive influence was observed in the post-hoc analysis of patients with severe deafness (Mader et al., 2018).

Although the quantity of FDA-approved clinical studies of relevant drugs is gradually mounting, current clinical findings affirming the clinical advantages of hydrogel delivery systems over traditional tympanic membrane injection drugs (such as glucocorticoid tympanic membrane injection) are still inconclusive. Further optimization and perfection are still warranted.

## 5 Conclusion

In light of the ability of hydrogel delivery systems to reversibly block Eustachian tubes, they have been increasingly popular in the development of intratympanic injections for the treatment of inner ear diseases, especially environment-responsive hydrogels. The advancement of these novel nanodrugs is anticipated to address the long-standing challenge of inner ear delivery in clinical treatments. However, the development of the hydrogel inner ear drug delivery system still faces many technical bottlenecks that need to be solved urgently. Material science research on hydrogel structure remains incomplete, encompassing the enhancement of biomaterials, admixture of supplements, classification and mode of cross-linking, and other related aspects. These properties enormously affect the physical and chemical properties of hydrogels in the auditory system, such as phase transition time and sustained release period. Meanwhile, the real-time monitoring of hydrogel clearance in vivo are still under tapped. Current studies mainly employed glucocorticoids for inner ear administration, and the efficiency of macromolecular inner ear administration is still not ideal.
